# Characterization of the Immune Cell Infiltration Landscape of Thyroid Cancer for Improved Immunotherapy

**DOI:** 10.3389/fmolb.2021.714053

**Published:** 2021-11-01

**Authors:** Jing Gong, Bo Jin, Liang Shang, Ning Liu

**Affiliations:** ^1^ Department of Geriatrics, The First Hospital of China Medical University, Shenyang, China; ^2^ Department of Medical Oncology, Key Laboratory of Anticancer Drugs and Biotherapy of Liaoning Province, Liaoning Province Clinical Research Center for Cancer, The First Hospital of China Medical University, Shenyang, China; ^3^ Innovative Research Center for Integrated Cancer Omics, The Second Affiliated Hospital of China Medical University, Shenyang, China; ^4^ Department of Pancreatic and Biliary Surgery, The First Affiliated Hospital of China Medical University, Shenyang, China

**Keywords:** landscape, thyroid cancer, immunotherapy, infiltration, prediction, prognosis

## Abstract

Within the endocrine system, thyroid cancer (THCA) is the most typical malignant tumor. Tumor-infiltrating immune cells play vital roles in tumor progression, recurrence, metastasis as well as response to immunotherapy. However, THCA’s immune infiltrative landscape is still not clarified. Therefore, we utilized two statistical algorithms to investigate the immune cell infiltration (ICI) landscape of 505 THCA samples and defined three ICI immune subtypes. The ICI scores were calculated using principal-component analysis. Increased tumor mutation burden (TMB) and immune-related signaling pathways were associated to a high ICI score. The high ICI score group indicated a relatively longer overall survival (OS) than the low ICI score group. Most immune checkpoint-related and immune activation-related genes were considerably upregulated in the ICI high group, which indicates stronger immunogenicity and a greater likelihood of benefiting from immunotherapy. In two cohort studies of patients receiving immunotherapy, high-ICI-score group showed notable therapeutic effects and clinical advantages compared to those with lower ICI scores. These results demonstrate that ICI score acts as an effective prognostic indicator and predictor of response to immunotherapy.

## Introduction

The most common malignant tumor of the endocrine system is thyroid cancer (THCA). As tumor detection has advanced, the global incidence of THCA has risen rapidly. Younger people and women are more likely to suffer from it (La Vecchia et al., 2015; Du et al., 2018; Siegel et al., 2020). There are four major pathological subtypes of thyroid cancer: papillary thyroid carcinoma, follicular thyroid carcinoma, medullary thyroid carcinoma, and undifferentiated carcinoma. The prognoses vary greatly (Cabanillas et al., 2016). Traditional treatments including chemotherapy and radiotherapy cannot improve the therapeutic effect of locally advanced or metastatic thyroid cancer, which makes the development of effective treatments crucial.

The tumor microenvironment (TME) of thyroid cancer is rich in immune cells, which indicates that it is an ideal candidate for immunotherapy. And certain immune properties have been shown to affect the prognosis of thyroid cancer (Ferrari et al., 2019). Extensive researches on the immune microenvironment of thyroid cancer have shown that components of an individual’s immune system were closely related to the occurrence, invasion, and metastasis as well as the therapeutic response to immunotherapy (Xie et al., 2020; Yin et al., 2020). A new clinical experiment showed that tumor immune cell infiltration (ICI) in thyroid cancer is related to sensitivity to immunotherapy and a better prognosis (Na and Choi, 2018). In papillary thyroid carcinoma, tumor-associated macrophages (TAMs) are correlated to lymph node metastasis, increased tumor size, and a diminished survival rate (Kim et al., 2013; Fang et al., 2014). Increased regulatory T cell (Treg) infiltration suggests a positive correlation with disease stage, whereas natural killer (NK) cell infiltration is adversely associated with disease stage (Gogali et al., 2012). Additionally, thyroid cancer cells can produce a variety of cytokines and chemokines, which can promote the tumor progression. Reducing the concentration of cytokines and chemokines in the tumor microenvironment produces therapeutic benefits (Coperchini et al., 2019). An improved understanding of the molecular and immunological properties of the THCA tumor microenvironment can be used to aid immunotherapy.

We collected The Cancer Genome Atlas (TCGA)-THCA datasets, evaluating the immune microenvironment and immune cell infiltration in THCA using two calculation tools, CIBERSORT, and ESTIMATE. According to the TCGA-THCA immunophenoscores (IPSs), we identified the relevant genes to establish an immune cell infiltration (ICI) scoring model and verified it using the TCGA-THCA dataset. In summary, this ICI score system can be an effective prognostic biomarker and predictor for the valuation of THCA immunotherapy response.

## Methods

### Obtaining Expression Profile Data and Clinical Data

The overall analysis ideas of this research are shown as follows. First, download THCA expression profile data as well as follow-up clinical evidences based on the TCGA database (https://portal.gdc.cancer.gov/). The RNA sequence data of TCGA-THCA is processed in the following steps: 1) Remove samples which did not include subsequent clinical information; 2) Remove samples with no survival period and survival status; 3) Convert the probe to Gene Symbol; 4) One probe corresponds to several genes, remove the probe; 5) Use the median value for the expression of multiple Gene Symbols. The pre-processed TCGA-THCA data has a total of 505 tumor samples, and the clinical statistics of the samples are shown in Table 1.

**TABLE 1:** Clinical information of TCGA-THCA datasets.

		TCGA-THCA
Survial		
OS	status_0	489
	status_1	16
histological_type	Classical.usual	361
	Follicular	99
	Tall.Cell	36
	Others	9
Age		
	Age>60	113
	Age< = 60	392
Gender		
	Female	367
	Male	138
Stage		
	Stage_I	284
	Stage_II	52
	Stage_III	112
	Stage_IV	55
	Stage_un	2
Mstage	M0	286
	M1	8
	MX	210
	M_un	1
Nstage	N0	227
	N1	229
	NX	49
Tstage	T1	142
	T2	165
	T3	174
	T4	22
	TX	2
RT (Radiation therapy)	YES	310
	NO	177
	UN	18
TMT (Targeted molecular therapy)	YES	5
	NO	94
	UN	406

### Tumor Immunophenoscore (IPS Database)

Tumor immunophenoscore (IPS) comes from the website of The Cancer Immunome database (TCIA) (https://tcia.at/patients). It is in view of the characteristics of tumor-infiltrating immune cells, acting as a bridge between immune cell infiltrating subtypes and immune gene subtypes. (Charoentong et al., 2016).

### Consensus Clustering Analysis of Immune Cell Infiltration

22 different immune cells infiltration level in THCA (naïve B cells, memory B cells, plasma cells, CD8^+^ T cells, naïve CD4^+^ T cells, inactivated and activated memory CD4^+^ T cells, T follicular helper cells, Tregs, gamma delta T cells, resting and activated NK cells, monocytes, M0, M1, and M2 macrophages, resting and activated dendritic cells, resting and activated mast cells, eosinophils, and neutrophils) was quantified utilizing the CIBERSORT R package, based on the LM22 signature and 1,000 permutations. We use ESTIMATE R package to estimate the degree of immune infiltration and matrix purity scores in each THCA sample. “Pam” method according to Euclid and Ward’s linkages was utilized, and executed by “ConsensuClusterPlus package” R package. Unsupervised clustering was performed, and researchers repeated the clustering 1,000 times to ensure the classification’s stability.

### Differential Expression of Genes Associated With the Tumor IPS

The tumor samples were divided into two groups according to the optimal density gradient threshold of tumor immunophenoscore (IPS) related to survival with the method of R Survminer package. The differential expression of genes was analyzed between the TCGA-THCA tumor sample with different score groups through specifying the cutoff value 0.05 (adjusted) and | log2 (Fold Change) | >1 using the limma R package. Furthermore, the genes which are positively related to the consistent classification result are called immune genotype-related feature A, and the remaining genes are called for feature B.

### Dimension Reduction of Gene Features and Construction of ICI Score

In order to measure the immune cell infiltration in tumors through gene expression, this study constructed a tumor immune cell infiltration score (ICI scores) model based on feature A and feature B gene sets related to immune gene subtypes. First of all, we conduct the Boruta algorithm to reduce dimension of the feature A and B genes. After reduction, ICI score A and B which were defined by ICI feature gene A and B were evaluated by the principal component analysis (PCA). Thirdly we construct an immune cell infiltration score (ICI scores) model, and the calculation formula is as follows (Sotiriou et al., 2006):

ICI_scores=∑PC1(A)−∑PC1(B)

### Collection of Somatic Alteration Data

Mutation data of TCGA-THCA were downloaded from the TCGA (https://www.cancer.gov/tcga/). We used the Survminer R package to calculate the optimal density gradient threshold related to the tumor mutation burden (TMB) and survival and categorized the samples into high and low tumor TMB groups. Implementing the maftool R package, the mutation frequencies of the top 30 driver genes in different immune cell infiltration score (ICI scores) groups were compared.

### Acquisition of Immunotherapy Data Sets

To explore the effectiveness of ICI score in predicting the benefit of immunotherapy treatment, we downloaded the expression profile data and clinical materials of the IMvigor210 cohort (http://research-pub.gene.com/IMvigor210CoreBiologies/), the ICI scoring model is used to divide all samples into a high and a low scoring group. Similarly, the GSE78220 data set was downloaded from GEO and analyzed accordingly.

### Statistical Analyses

All the statistical comparisons involved in this study and the hypothesis testing of the significance of differences between groups are based on the statistical analysis method of R 3.6.

## Results

### The Immune Cell Infiltration Landscape

**FIGURE 1:**
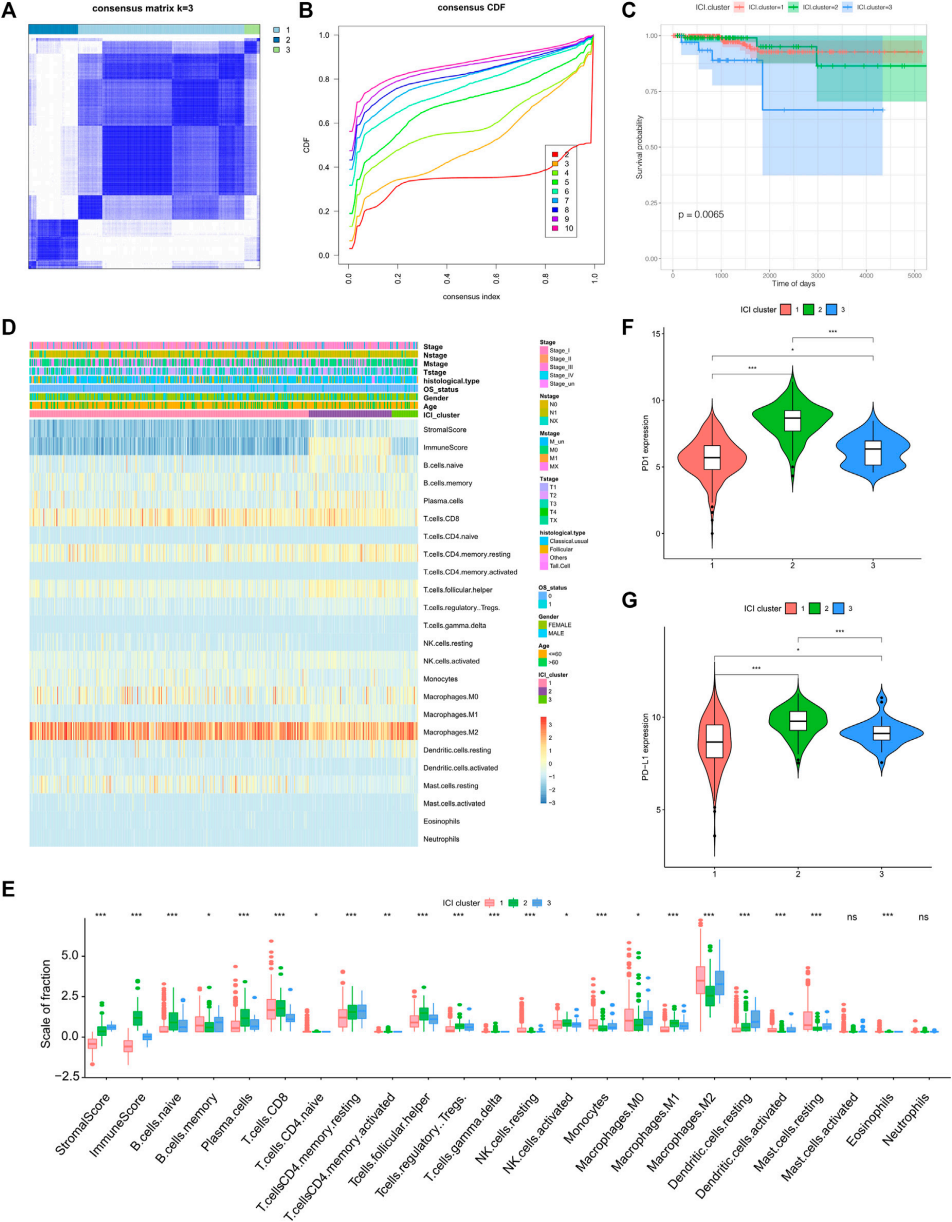
Identification of tumor immune subtypes and characteristics of immune infiltration. **(A)** Clustering result when k = 3; **(B)** CDF curve distribution of consistent clustering; **(C)** Survival curve when K = 3; **(D)** Heat map of immune cell infiltration characteristics; **(E)** Box diagram of differences in immune cell infiltration characteristics among the three ICI subtypes; **(F)** The expression difference of PD1 among the three ICI subtypes; **(G)** the expression difference of PD-L1 among the three ICI subtypes.

For each sample in the TCGA-THCA dataset, 22 types of immune cell’s infiltration statuses (naïve B cells, memory B cells, plasma cells, CD8^+^ T cells, naïve CD4^+^ T cells, resting and activated memory CD4^+^ T cells, T follicular helper cells, Tregs, gamma delta T cells, resting and activated NK cells, monocytes, M0, M1, and M2 macrophages, resting and activated dendritic cells, resting and mast cells, eosinophils, and neutrophils) were recorded (Supplementary Table S1). From this, a cluster correlation and cumulative distribution function (CDF) curve were calculated (k = 3) and used for stable Immune Cell Infiltration (ICI) subtype classification (Figures 1A,B). Of the three main subtypes, ICI3 had the worst prognosis and a median OS of 937 days, whereas ICI1/2 had a mean OS of 1,017 days (p = 0.0065, Figure 1C).

To better clarify the inherent biological causes of the different clinical phenotypes, the TMEs of the three molecular subtypes were compared and visualized using heat maps (Figure 1D,E). The ICI3 samples were characterized by high stromal scores, low infiltration of CD8^+^ T cells, and greater infiltration of static memory CD4^+^ T cells and active dendritic cells. Subtypes ICI1 and ICI2, which had better prognoses, were characterized by lower stromal scores, greater CD8^+^ T cell infiltration, lower infiltration of Tregs, inactive memory CD4^+^ T cells and active dendritic cells.

In addition to analyzing immune cell infiltration, the expression levels of two important immune checkpoints (PD1 and PD-L1) in each ICI subtype were further analyzed. One of the characteristics of the ICI3 subtype was significantly lower PD1/PD-L1 expression, while the ICI2 subtype had higher PD1/PD-L1 expression. To assess the statistical significance of the differences in immune infiltration and PD1/PD-L1 expression among the ICI subtypes, the Kruskal-Wallis test and a hypothesis test were used. As shown in Figures 1F,G, the differences in PD1/PD-L1 expression and the infiltration of most immune cell types were statistically significant.

### Immune Gene Expression Subtypes

In order to display different biological characteristics of immune phenotype, the tumor immunophenoscores (IPSs) samples in the TCGA-THCA dataset were collected from The Cancer Immunome database (TCIA), as shown in Supplementary Table S2. The Survminer R package was used to calculate the optimal density gradient threshold for the IPSs. A high and a low group of IPS with a threshold of seven were presented from TCGA-THCA tumor samples. The survival of high IPS group was much longer than the other group significantly (Supplementary Figure S1).

**FIGURE 2:**
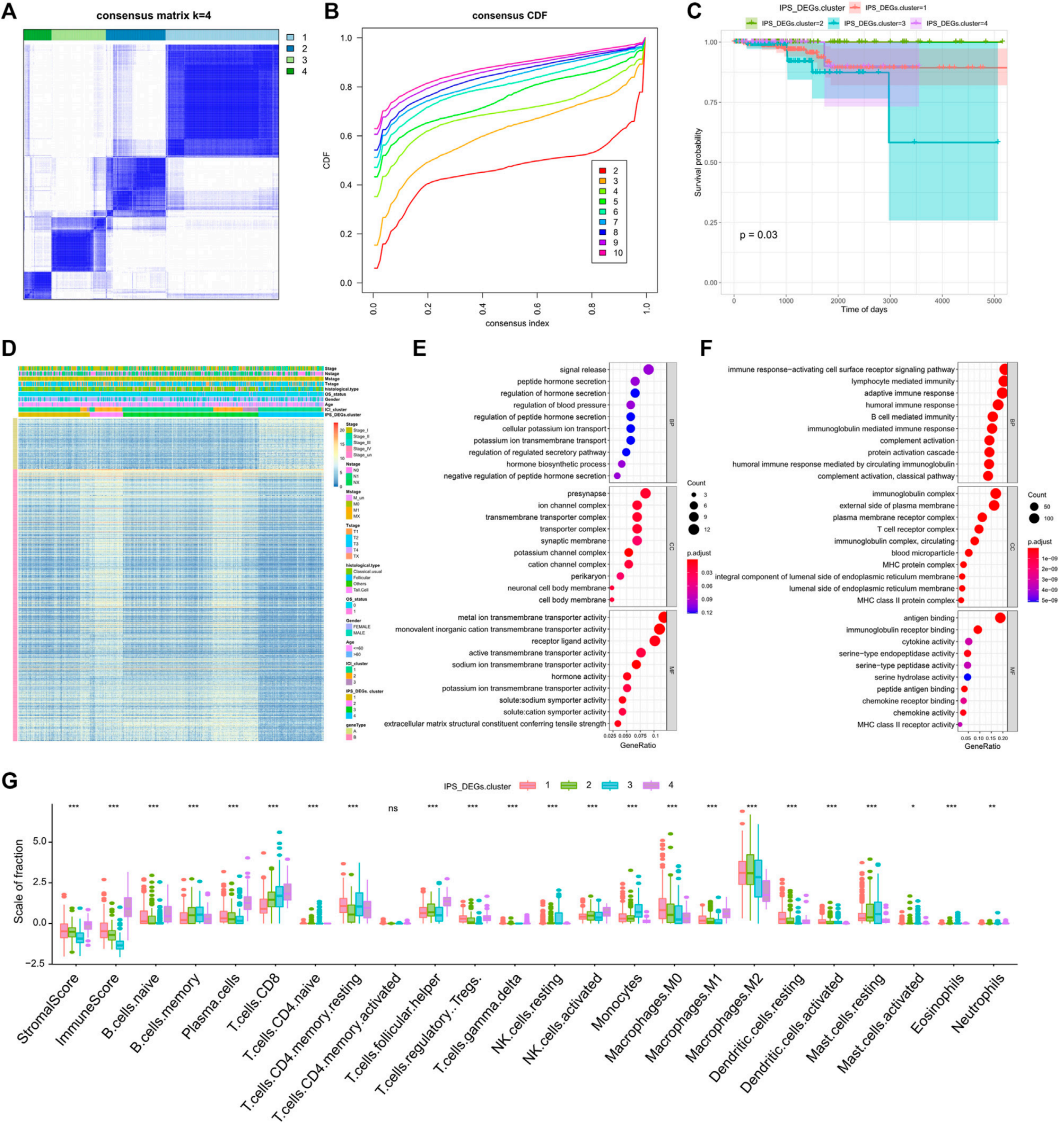
Consistent clustering of differentially expressed genes related to tumor immune antigenicity. **(A)** Clustering results when k = 4; **(B)** CDF curve distribution of consistent clustering; **(C)** Survival curve of molecular subtypes when k = 4; **(D)** Heat map of immune gene subtypes; **(E)** Functional enrichment of immune gene subtype related features A gene; **(F)** Functional enrichment of immune gene subtype related features B gene; **(G)** Differences in infiltration characteristics of immune cells between immune gene subtypes.

We analyzed differential gene expression (DEG) of different IPS group utilizing the limma R package with the set screening threshold changed (p < 0.05 and | log2 (Fold Change) | >1). One thousand two-hundred and ten differentially expressed genes (Supplementary Table S3) were identified, of which 1,030 genes were actively expressed in the high IPS group and 180 genes were greatly expressed in the low IPS group. We then performed unsupervised clustering of 1,210 differentially expressed genes related to IPS (IPS_DEGs) with the ConsensuClusterPlus R package. Finally, according to the cluster correlation and CDF curve, the TCGA-THCA tumor samples were divided into four immune gene expression subtypes, IPS_DEGs Clusters 1-4 (Figures 2A,B). There were clear differences in survival between the different immune gene expression subtypes (log rank test, p = 0.03, Figure 2C). Gene Type A includes 191 gene signatures which had a positive association with immune gene subtypes, while the remaining 1019 IPS_DEGs were represented by Gene Type B (Supplementary Table S4)

We revealed the heatmap of gene expression for each ICI subtype, IPS_DEGs Cluster, and Gene Type (Figure 2D) and implemented gene ontology (GO) functional enrichment study on Gene Type A and B genes using the clusterProfiler R package. A bubble map was used to display the first 10 pathways enriched in three groups of different functions (Biological Process, Cellular Component, Molecular Function), as shown in Figures 2E,F. Most of the enriched pathways were related to immunobiological processes.

We examined the 22 immune infiltration cells in the four IPS_DEG subtypes and found considerable differences between different subtypes (Figure 2G). We also observed great differences in PD1/PD-L1 expression. IPS_DEGs clusters 1, 2, and 4 were related to greater PD1/PD-L1 expression, while IPS_DEGs cluster 3 had the lowest PD1/PD-L1 expression (Supplementary Figure S2A, B). This low PD1/PD-L1 expression indicates a lack of sensitivity to immunotherapy, which is related to a poor prognosis. The consistent associations between immune cell infiltration characteristics and the prognosis of different gene expression subtypes indicate that the classification strategy for the immune cell subtype is scientific and reasonable.

### ICI Score Construction

**FIGURE 3:**
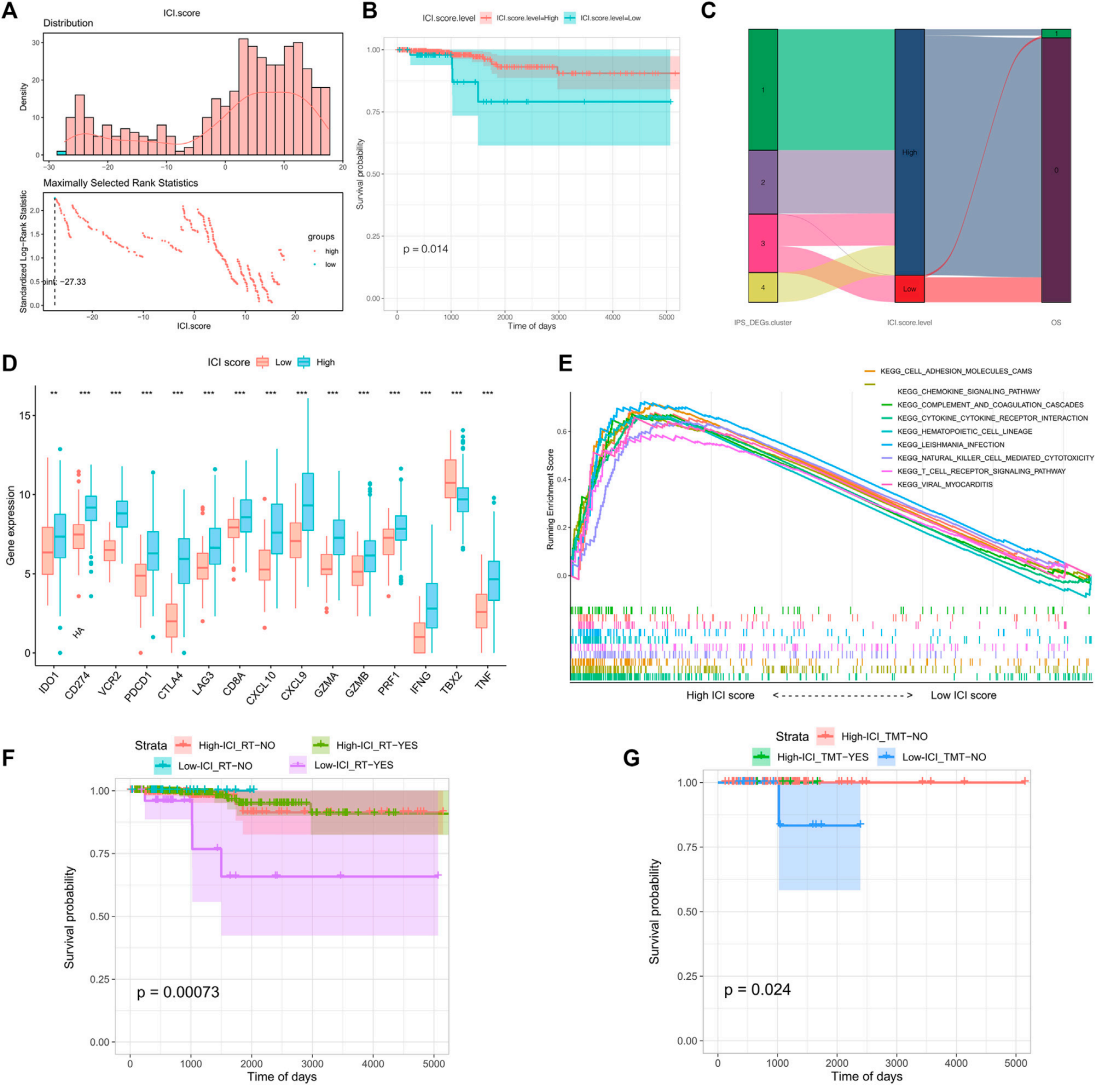
Grouping and model construction of tumor immune cell infiltration scores (ICI scores). **(A)** Distribution **(above)** and the selection of the best density gradient points **(below)** of immune cell infiltration score (ICI scores); **(B)** survival curve between high and low ICI scores; **(C)** Sankey diagram of immune gene subtypes, ICI scores grouping and survival status; **(D)** Differences in immune checkpoints and immune activation gene expression between ICI scores groups; **(E)** Gene set function enrichment of differentially expressed genes between ICI scores groups; **(F)** Survival curve between radiotherapy and ICI scores groups; **(G)** Survival curve received therapy in different ICI scores.

To measure the immune cell infiltration in thyroid tumors using differential expression genes, we constructed a tumor immune cell infiltration (ICI) score model based on the type A and type B gene sets associated with immune gene subtypes. The Survminer R package was utilized to calculate the most accurate density gradient threshold related to ICI scores and survival. The cutoff value was -27.33, which divided the TCGA-THCA tumor samples into high and low ICI score groups. The high ICI score group manifested longer OS than the low ICI score group in Figures 3A,B (p = 0.014). A Sangji diagram was adopted to visualize the relations between the immune gene expression subtypes, ICI score groups, and survival statuses (Figure 3C).

Before the prognostic value of ICI score could be assessed for the TCGA-THCA cohort, it was necessary to analyze the immune activity and tolerance conditions within different groups. We chose CD274, CTLA4, HAVCR2, IDO1, LAG3, and PDCD1 as immune checkpoint features, and CD8A, CXCL10, CXCL9, GZMA, GZMB, IFNG, PRF1, TBX2, and TNF as immune activation-related genes (Zhang et al., 2020a). Except for IDO1, TBX2, and TNF, expression of most genes related to immune checkpoints and immune activity was significantly increased in the high ICI score group (Figure 3D). We conducted gene set enrichment analysis (GSEA) to make an in-depth exploration of the biological differences between the two ICI score groups. The top ten enriched pathways were cell adhesion molecules (CAMs), chemokine signaling pathways, complement and coagulation cascades, cytokine receptor interactions, hematopoietic cell lineage, leishmania infection, NK cell mediated cytotoxicity, T cell receptor signaling, and viral myocarditis (Figure 3E). We also evaluated the impact of radiotherapy and targeted therapy on the prognosis of each ICI subgroup. The patients of high ICI score group had a better survival compared with the other group no matter what kind of treatment they received (Figures 3F,G).

### Somatic Variants in Different ICI Groups

A large body of data suggests that the tumor mutation burden (TMB) may forecast the response to immunotherapy. Thus, researching on the relationship between TMB and ICI score to clarify the genetic characteristics is crucial. We first used the Survminer R package to calculate the optimal density gradient threshold for TMB and survival. A score of 0.42 was the threshold that divided the TCGA-THCA tumor samples into two groups: high TMB group and low TMB group. There were significant differences in survival between the two groups, as shown in Supplementary Figure S3.

**FIGURE 4:**
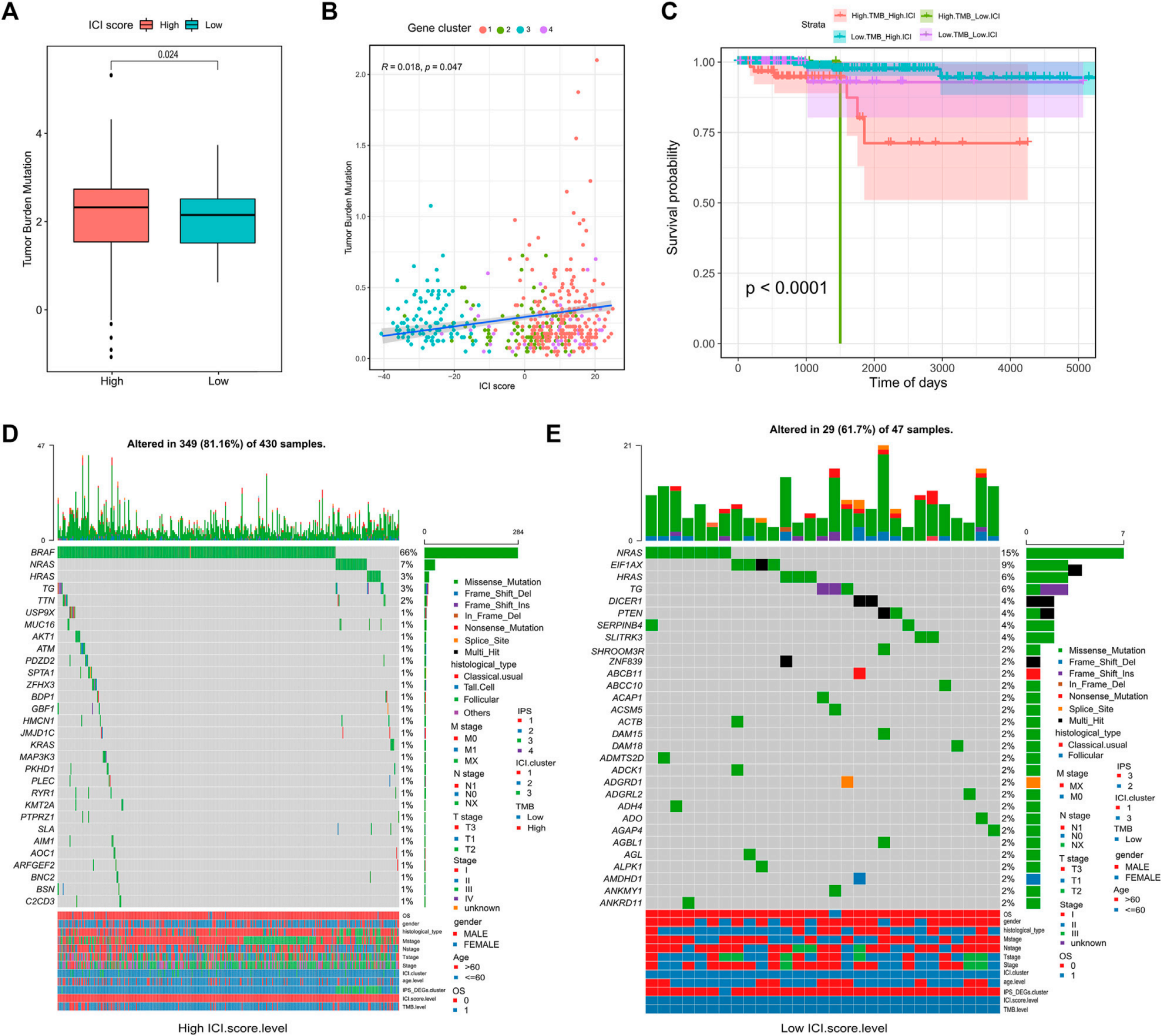
Correlation between ICI scores and Somatic Variants. **(A)** Tumor mutation burden **(TMB) **between high and low ICI scores groups; **(B)** Association between ICI scores and tumor mutation burden **(TMB);** **(C)** Survival curve between tumor mutation burden **(TMB)** and ICI scores groups; **(D)** waterfall chart of gene mutation distribution in tumors of high ICI scores group; **(E)** waterfall chart of gene mutation distribution in tumors of low ICI scores group.

We first conducted a comparison of the TMB in the high and low ICI score groups. According to Figure 4A, TMB were significantly higher in the high ICI score group (Wilcoxon test, p = 0.024). Further correlation analysis showed a significant positive relationship between ICI score and TMB score (Spearman coefficient: R = 0.018, p = 0.047; Figure 4B). However, the prognosis of patients with high TMB performed worse prognosis compared with low TMB patients, which was conflicted with the prognoses based on ICI scores. Therefore, in the prognostic stratification of THCA, we further analyzed the synergy of TMB and ICI score. According to the TMB, the stratified survival analysis showed that either in the high or low TMB subgroups, the prognosis of patients with the high ICI score were always better than the low ICI score group (p <0.05; Figure 4C). This indicates that TMB status does not impede predictive effects of ICI scores. Accordingly, the above-mentioned results demonstrated that the ICI score may be a potential predictor of patient prognosis independent of TMB and that it is an effective predictor of response to immunotherapy.

In addition to overall TMB, the distribution of somatic variation in THCA driver genes between different ICI subgroups was further analyzed. And the 30 drive genes with the most significant change frequencies were compared (Figures 4D,E). We analyzed the mutation annotation files from the TCGA-THCA data and found huge variations in the mutation profiles of the high and low ICI subgroups. These findings may provide new insight for studying the tumor ICI composition and understanding mutational mechanisms affecting immune checkpoint inhibitor therapy.

### The Value of ICI Scores in Predicting Response to Immunotherapy

**FIGURE 5:**
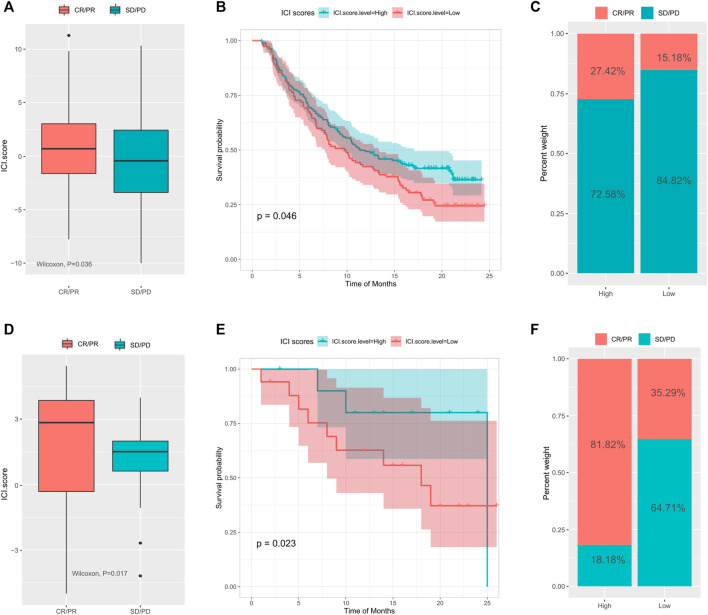
The role of tumor immune cell infiltration scores in the prediction of immunotherapy. **(A)** The difference of ICI scores between different treatment response groups in the IMvigor210 cohort; **(B)** the survival curve between the high and low ICI scores groups in the IMvigor210 cohort; **(C)** the difference of response between different ICI scores in the IMvigor210 cohort; **(D)** Difference of ICI scores between different treatment response groups in the GSE78220 cohort; **(E)** survival curve between different ICI score groups in the GSE78220 cohort; **(F)** difference of proportion of response between different ICI score groups in the GSE78220 cohort.

Immune checkpoint blockade therapy has emerged to treat cancer by blocking T cell inhibition pathways. To explore the relationship between ICI scores and response to immunotherapy, we analyzed expression profile data and clinical materials from the IMvigor210 cohort. All samples were defined within high and low ICI score groups using the ICI scoring model. Our results showed that in the IMvigor210 cohort, high ICI scores were associated with greater objective response to anti-PD-L1 treatment (Wilcoxon test, p = 0.036, Figure 5A) and longer survival (log rank test, p = 0.046, Figure 5B). Patients in the high ICI score group had higher objective responses to anti-PD-L1 treatment than (Wilcoxon test, p < 0.01; Figure 5C). The GSE78220 cohort in GEO, which received different types of immunotherapies, including cytokines, vaccines, and checkpoint blockers, showed similar findings (Wilcoxon test, p = 0.017; Figure 5D; log rank test, p = 0.023, Figure 5E; chi-square test, p0.01, Figure 5F). Overall, these findings pointed out that the ICI score may predict response to immunotherapy.

## Discussion

Immunotherapy has shown amazing efficacy against a variety of malignant tumors, but for thyroid cancer it is still in exploratory stages. An early clinical trial showed that the PD-1 inhibitor pembrolizumab may be safe and effective in treating PD-L1-positive thyroid cancer. Studies have also shown a positive therapeutic effect of immunotherapy for patients with undifferentiated thyroid cancer (Capdevila et al., 2020; Ma et al., 2020). Combination therapies, including immune checkpoint inhibitors and tyrosine kinase (TK) or serine/threonine protein kinase B-Raf (BRAF) inhibitors, have shown significant potential for the treatment of advanced thyroid cancer (Iyer et al., 2018; Varricchi et al., 2019). Finding predictive indicators for evaluating the efficacy and prognosis of immunotherapy in thyroid cancer has now become a problem that needs to be solved urgently.

We analyzed the ICI of 505 THCA samples and divided the samples into three immune subtypes. Activated CD8^+^ T lymphocytes are critical for adaptive immune defense and kill cancer cells through a variety of mechanisms; they are generally considered the main cytotoxic lymphocytes exerting anti-tumor effects (Farhood et al., 2019; van der Leun et al., 2020). CD8^+^ T cell infiltration into the tumor indicates better prognosis (Bai et al., 2017; Ye et al., 2019; E et al., 2018). Tumor-associated dendritic cells (DCs) have functional defects, which can cause cancer immunosuppression (Veglia and Gabrilovich, 2017). In the TME, there are DCs with impaired antigen cross-presentation, and this can lead to changes in the activation and maintenance of anti-tumor immunity and promote tumor progression (Liu and Cao, 2015). In the present study, the ICI3 subtype with poor prognosis was characterized by low CD8^+^ T cell infiltration, high infiltration of static memory CD4^+^ T cells and active DCs, and a high stromal score. The ICI1 and ICI2 subtypes, which had good prognoses, were characterized by high infiltration of CD8^+^ T cells, low infiltration of regulatory T cells and activated DCs, and a low stromal score; this was consistent with previous results. Our survival analysis showed a higher OS of the high ICI score group, which is in agreement with previous studies on TME and patient prognosis (Li et al., 2019). We also found that most immune checkpoint-related and immune activation-related genes were upregulated in the high ICI score group, which indicates better immunogenicity and greater benefit from immunotherapy.

The gene set enrichment analysis (GSEA) was applied to study the biological distinctions between the two ICI score subgroups and to obtain the following immune-related pathways: chemokine signaling, cytokine receptor interactions, NK cell-mediated cytotoxicity, and T cell receptor signaling. Chemokines play important immunomodulatory roles by regulating cell proliferation, migration, activation, differentiation, and homing (Nagarsheth et al., 2017). NK cells mediate anti-tumor immunity through cytotoxicity and cytokine secretion (Müller-Hermelink et al., 2008; Gross et al., 2013). The TME of thyroid cancer produces soluble modulators that negatively regulate the maturation, proliferation, and effector functions of NK cells (Melaiu et al., 2020). It has also been shown that TCR signaling shapes the TCR composition of Foxp3+ regulatory T cells (Burchill et al., 2008). Research on cytokine receptor signaling pathways in thyroid cancer has thus far focused on the role of IL-1 family members (Mantovani et al., 2019).

Previous work has suggested that TMB is a prognostic marker and effective predictor of response towards immunotherapy in many cancers (Marabelle et al., 2020). The correlation between TMB and immune infiltration can be used to further assess the response to immunotherapy for a variety of tumors more effectively, including melanoma, bladder urothelial cancer, head and neck cancer, renal clear cell carcinoma, endometrial cancer, and others. Low TMB is associated with reduced immune infiltration, suggesting a poor prognosis for patients (Zhang et al., 2019; Zhang et al., 2020b; Jiang et al., 2020; Kang et al., 2020; Jiang et al., 2021; Zhou et al., 2021). In this study, ICI scores showed significant differences in survival either in high TMB or low TMB subgroups. Moreover, regardless of the expression in TMB, the prognosis of patients in the high ICI score group is always better than that of the low ICI score group, which shows that the TMB status will not interfere with the prediction based on the ICI score. These findings indicate that ICI score and TMB represent different aspects of tumor immunobiology, and ICI score may be a potential biomarker independent of TMB and effective in predicting response to immunotherapy.

There is prior evidence for gene mutations associated with response or tolerance to immunotherapy. For example, POLE/POLD1 gene mutations are associated with microsatellite instability (MSI), mismatch repair (MMR), and TMB, and CMTM6 is related to PD-L1 expression and regulation of anti-tumor immunity (Burr et al., 2017; Yao et al., 2019). In the present study, there were significant differences in gene mutation profiles between the different ICI score groups; this may provide new insight for studying the ICI composition of thyroid tumors and its relationship to gene mutations.

To further evaluate the value of the ICI score for estimating patient responses to immunotherapy, the IMvigor210, and GSE78220 cohorts of patients receiving immunotherapy were analyzed. As seen from the results, patients of the high ICI score group possessed higher objective responses to anti-PD-L1 therapy and greater overall survival. These results suggested that the ICI score relates to immunotherapy response and is an effective predictor of immunotherapy efficacy. Patients with high ICI scores may benefit more from immunotherapy.

Considering the enriched chemokine signaling pathways in the high ICI score group, the patients may also benefit from chemokine suppression and immune checkpoint blockade therapies. Previous studies have shown that the CXCR1/2 inhibitor Reparixin affects the viability of various thyroid cancer cells (Liotti et al., 2017). A CXCR4 antagonist (AMD3100) has also been shown to have significant anti-tumor effects on BHP10-3 papillary thyroid carcinoma cells *in vivo* and *in vitro* (Jung et al., 2016). Chemokine antagonists are expected to be adjuvant in the thyroid cancer’s treatment.

In the present study, we adapted retrospective research and used public databases and bioinformatic approaches to discover potential biomarkers related to the development of THCA and the efficacy of immunotherapy. Due to tumor heterogeneity, future studies should include more clinicopathological characteristics to improve the accuracy of predictions. Further validation of our approach is still needed in a potential large-scale cohort of THCA patients treated immunotherapy.

In summary, we revealed the characteristics of ICI in THCA and established an ICI score which could accurately predict the patient’s prognosis and response to immunotherapy. The results exhibited a comprehensive outlook of the internal immune landscape of THCA tumors.

## Data Availability

The datasets presented in this study can be found in online repositories. The names of the repository/repositories and accession number(s) can be found in the article/Supplementary Material.
