# Supplementary figures and images for: Characterization of the Immune Cell Infiltration Landscape of Thyroid Cancer for Improved Immunotherapy
Source: Front Mol Biosci. 2021 Nov 1;8:714053. doi: 10.3389/fmolb.2021.714053 (PMC8591054; doi:10.3389/fmolb.2021.714053)

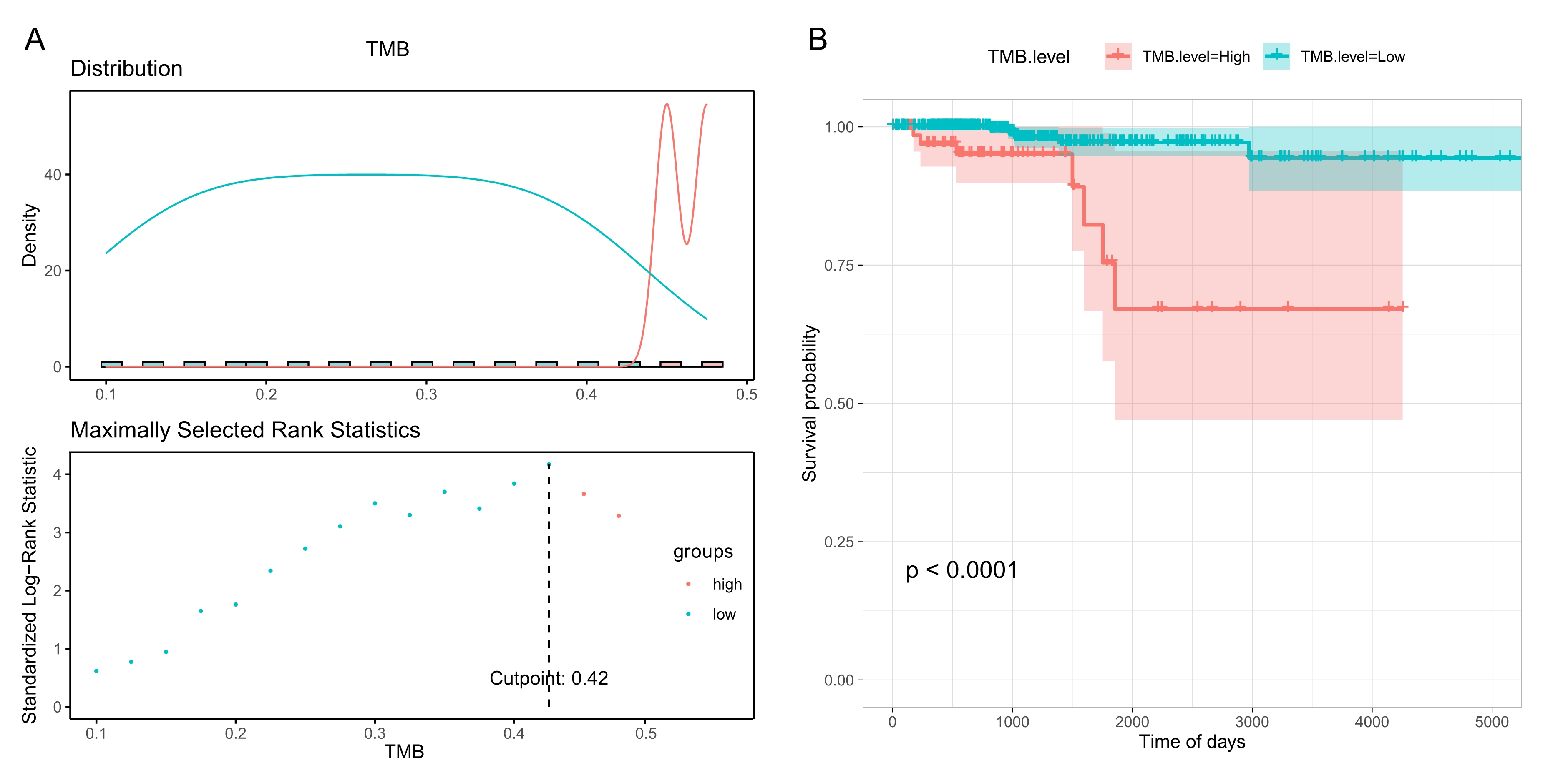

Supplement: Supplementary file 2 [file Image3.JPEG]

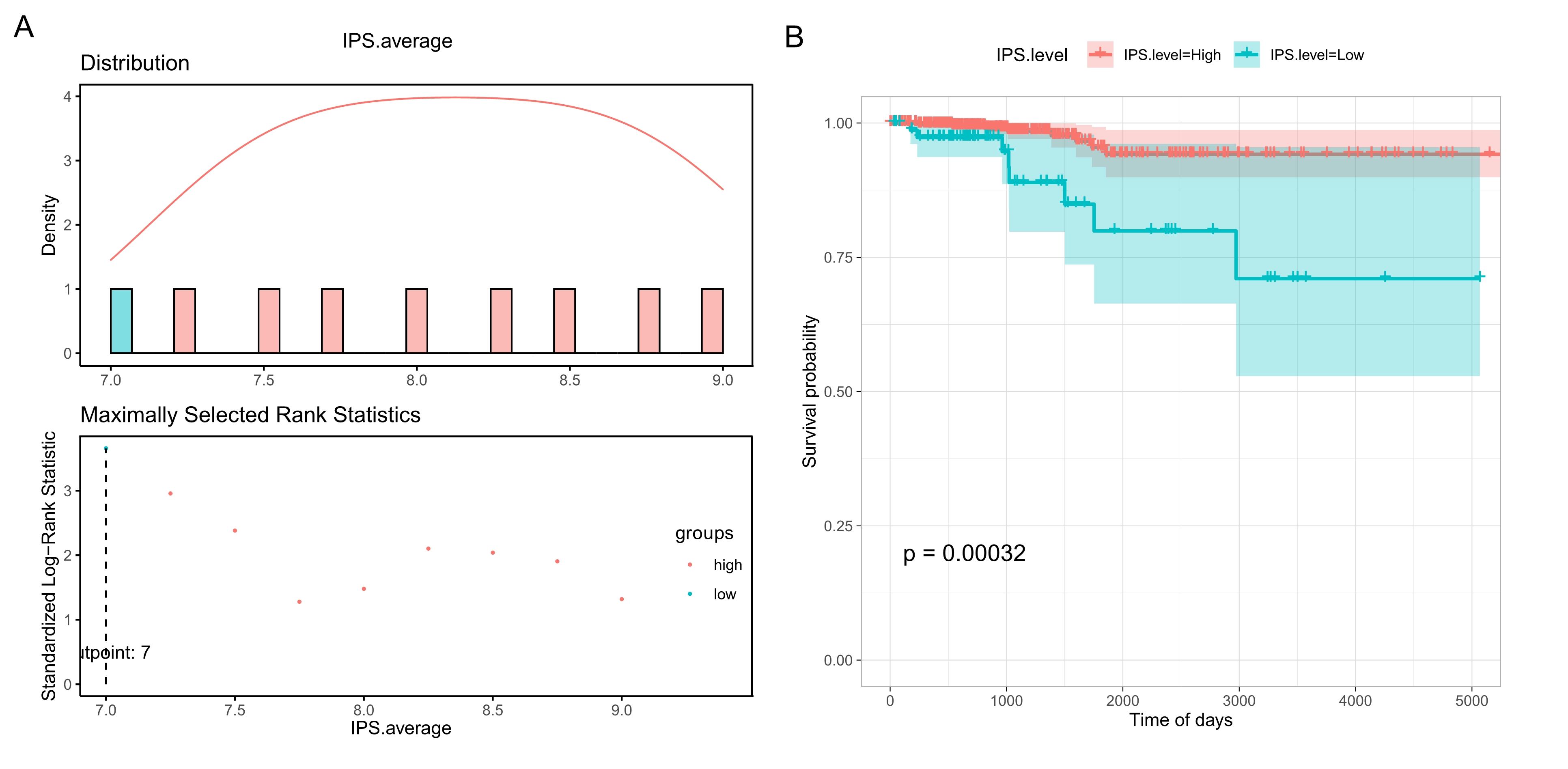

Supplement: Supplementary file 4 [file Image1.JPEG]

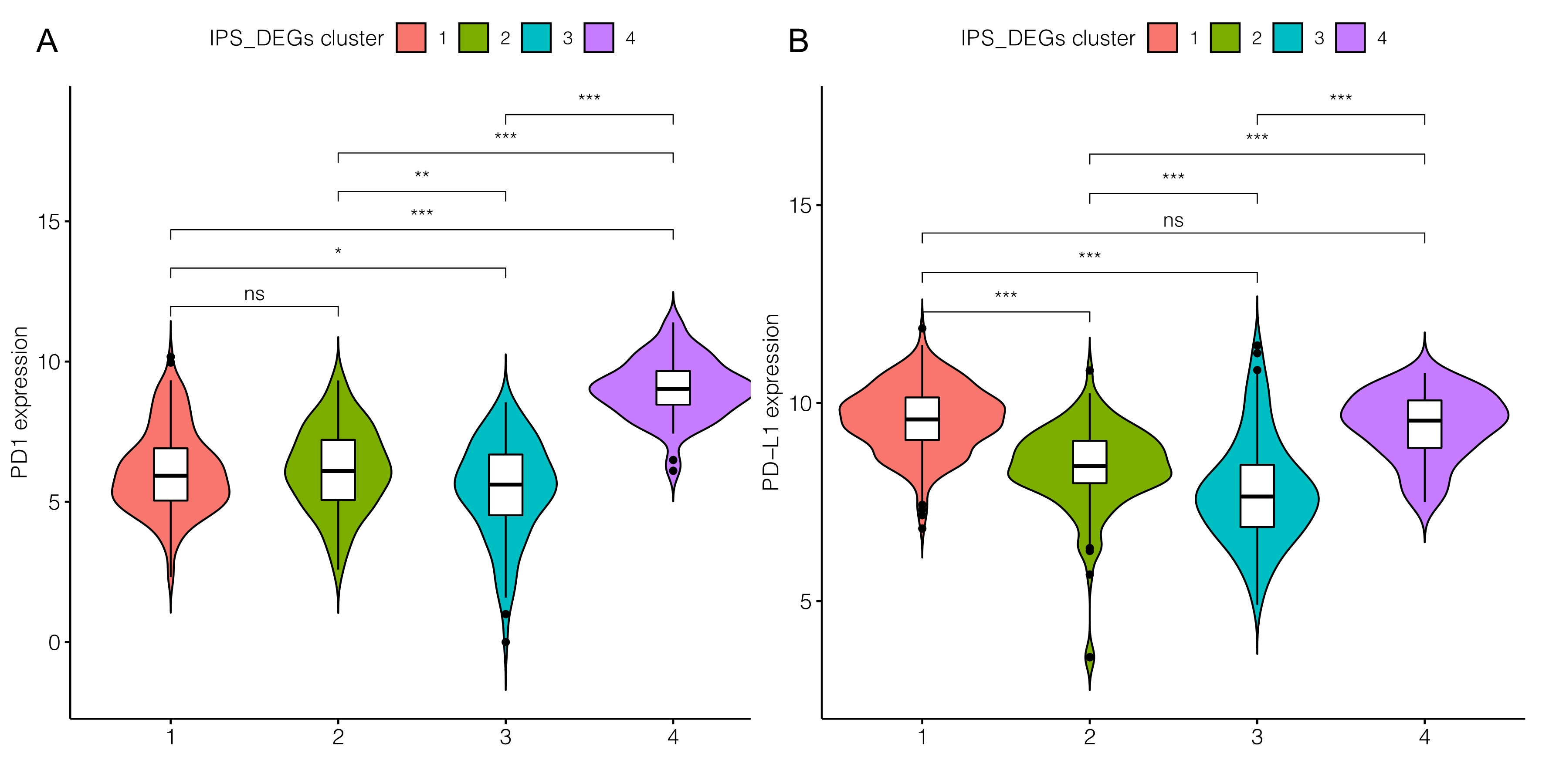

Supplement: Supplementary file 5 [file Image2.JPEG]
